# Colonization patterns of soil microbial communities in the Atacama Desert

**DOI:** 10.1186/2049-2618-1-28

**Published:** 2013-11-20

**Authors:** Alexander Crits-Christoph, Courtney K Robinson, Tyler Barnum, W Florian Fricke, Alfonso F Davila, Bruno Jedynak, Christopher P McKay, Jocelyne DiRuggiero

**Affiliations:** 1 Biology Department, The Johns Hopkins University, 3400 N. Charles Street, Mudd Hall, Baltimore, MD 21218, USA; 2 Institute for Genome Sciences, University of Maryland School of Medicine, Baltimore, MD, USA; 3 Space Science Division, NASA Ames, Mountain View, CA 94035, USA; 4 Department of Applied Mathematics and Statistics, Whiting School of Engineering, The Johns Hopkins University, Baltimore, MD, USA

**Keywords:** Soil microbial communities, Extreme environment, Arid soil, Atacama Desert, Desertification, High-throughput 16S rRNA sequencing

## Abstract

**Background:**

The Atacama Desert is one of the driest deserts in the world and its soil, with extremely low moisture, organic carbon content, and oxidizing conditions, is considered to be at the dry limit for life.

**Results:**

Analyses of high throughput DNA sequence data revealed that bacterial communities from six geographic locations in the hyper-arid core and along a North-South moisture gradient were structurally and phylogenetically distinct (ANOVA test for observed operating taxonomic units at 97% similarity (OTU_0.03_), *P* <0.001) and that communities from locations in the hyper-arid zone displayed the lowest levels of diversity. We found bacterial taxa similar to those found in other arid soil communities with an abundance of *Rubrobacterales*, *Actinomycetales*, *Acidimicrobiales*, and a number of families from the *Thermoleophilia.* The extremely low abundance of *Firmicutes* indicated that most bacteria in the soil were in the form of vegetative cells. Integrating molecular data with climate and soil geochemistry, we found that air relative humidity (RH) and soil conductivity significantly correlated with microbial communities’ diversity metrics (least squares linear regression for observed OTU_0.03_ and air RH and soil conductivity, *P* <0.001; UniFrac PCoA Spearman’s correlation for air RH and soil conductivity, *P* <0.0001), indicating that water availability and salt content are key factors in shaping the Atacama soil microbiome. Mineralization studies showed communities actively metabolizing in all soil samples, with increased rates in soils from the southern locations.

**Conclusions:**

Our results suggest that microorganisms in the driest soils of the Atacama Desert are in a state of stasis for most of the time, but can potentially metabolize if presented with liquid water for a sufficient duration. Over geological time, rare rain events and physicochemical factors potentially played a major role in selecting micro-organisms that are most adapted to extreme desiccating conditions.

## Background

Life can adapt to some of the harshest environments on Earth, from deep-sea hydrothermal vents to hypersaline lakes and acidic hot springs [1], but can life adapt to places where there is essentially no water and no nutrients? The Atacama Desert is one of the oldest and driest deserts in the world and its hyper-arid core has been described as ‘the most barren region imaginable’ [2,3]. While micro-organisms have been detected in the soil of the Atacama, little is known about the structure and composition of the microbial communities inhabiting its soil and the processes shaping the assembly of those communities.

The Atacama Desert stretches 600 miles along the Pacific Coast of Northern Chile (19º-27ºS) and its sedimentary records indicates semi-arid to hyper-arid climates from the Jurassic period (150 million years ago) to the present day, with extremely arid conditions arising in the Miocene (15 million years ago) [3-5]. The Atacama Desert owes its extreme aridity to a constant climate regime produced by a subtropical anticyclonic atmospheric subsidence - the Pacific Anticyclone. This is strengthened by the Humboldt Current - an upwelling, cold current along the west coast of South America - and the rain shadow effect from the Andean Cordillera to the East. The continentality effect that occurs when rain-bearing trade winds are blocked from penetrating continental interiors provides additional aridity in the desert [3,6]. Between parallels 22°S and 26°S is the hyper-arid core of the Atacama, one of the places with the lowest pluviometric activity in the world [2,6,7]. Long-term mean annual rainfall is only a few millimeters, with rain events typically occurring once per decade (http://www.meteochile.cl/; [2]). In addition, the high coastal mountains block the marine fog [2]. As a result, soil surfaces are bare with sparsely vegetated areas found only where groundwater discharges via localized springs [6]. Geological and soil mineral analyses of the hyper-arid core report organic material detected at trace levels, nitrate accumulation - probably of atmospheric origin - and highly oxidizing conditions [2,8,9]. Other unique features include very low erosion and the accumulation of halite, gypsum, anhydrite, and unusual salts such as perchlorates, nitrates, and iodates [5,10].

The extreme aridity of the Atacama Desert, together with broad daily temperature fluctuations and intense ultraviolet radiation, contribute to make the core of this desert an extreme habitat approaching ‘the dry limit of life on Earth’ [8]. Nevertheless, micro-organisms inhabit this extreme environment. Studies have reported low numbers of culturable bacteria in soil samples of the arid core, ranging from not detectable to 10^6^ CFU/g of soil, and reflecting a great spatial heterogeneity [8,9,11-15]. With the use of molecular methods, the subsurface layers of the hyper-arid core were shown to harbor a very limited microbial community dominated by *Gemmatimonadetes* and *Plantomycetes* bacteria and also including *Actinobacteria*, *Thermomicrobia*, and one member of the *Proteobacteria*[9,11,12]. These micro-organisms were identified in soil samples where no vascular plant has grown for millions of years and rain occurs only once every 20 to 50 years [8,9,11,12]. In less arid parts of the desert, soil bacterial communities were characterized by a high abundance of novel *Actinobacteria* and *Chloroflexi* taxa, and low levels of *Acidobacteria* and *Proteobacteria*[11,16]. Fungi cultured from samples collected in several locations of the Atacama were all spore-forming saprophytes, suggesting that they might not be indigenous to the desert but rather dispersed by wind [17].

Relatively diverse, photosynthetic-based microbial communities have been described colonizing diaphanous rocks and halite evaporites in various parts of the desert [18-23]. These endolithic and hypolithic habitats are considered environmental refuges for life in hot and cold deserts, and harbor highly specialized communities [24]. Questions still remain about the presence of any stable and functional microbial communities in the Atacama soil, due to its physical instability, low nutrient content, and non-translucent properties [8,23,25].

Here, we use a combination of geological analyses, mineralization experiments, and non-culture based high throughput molecular methods to determine whether the Atacama soil is at the dry limit for life. Our analyses of community structure and composition, from soil samples collected in the hyper-arid core and along a North-South moisture gradient, revealed a relatively simple ecosystem and indicated that salt content, together with water availability, significantly correlated with the diversity of microbial communities. Metabolic activity detected in soil samples with added moisture suggest that the soil community can be activated by rainfalls or heavy fog events, providing a means of adaptation to their extreme environmental conditions.

## Methods

### Sampling, soil geochemistry, and climate data

**Figure 1:**
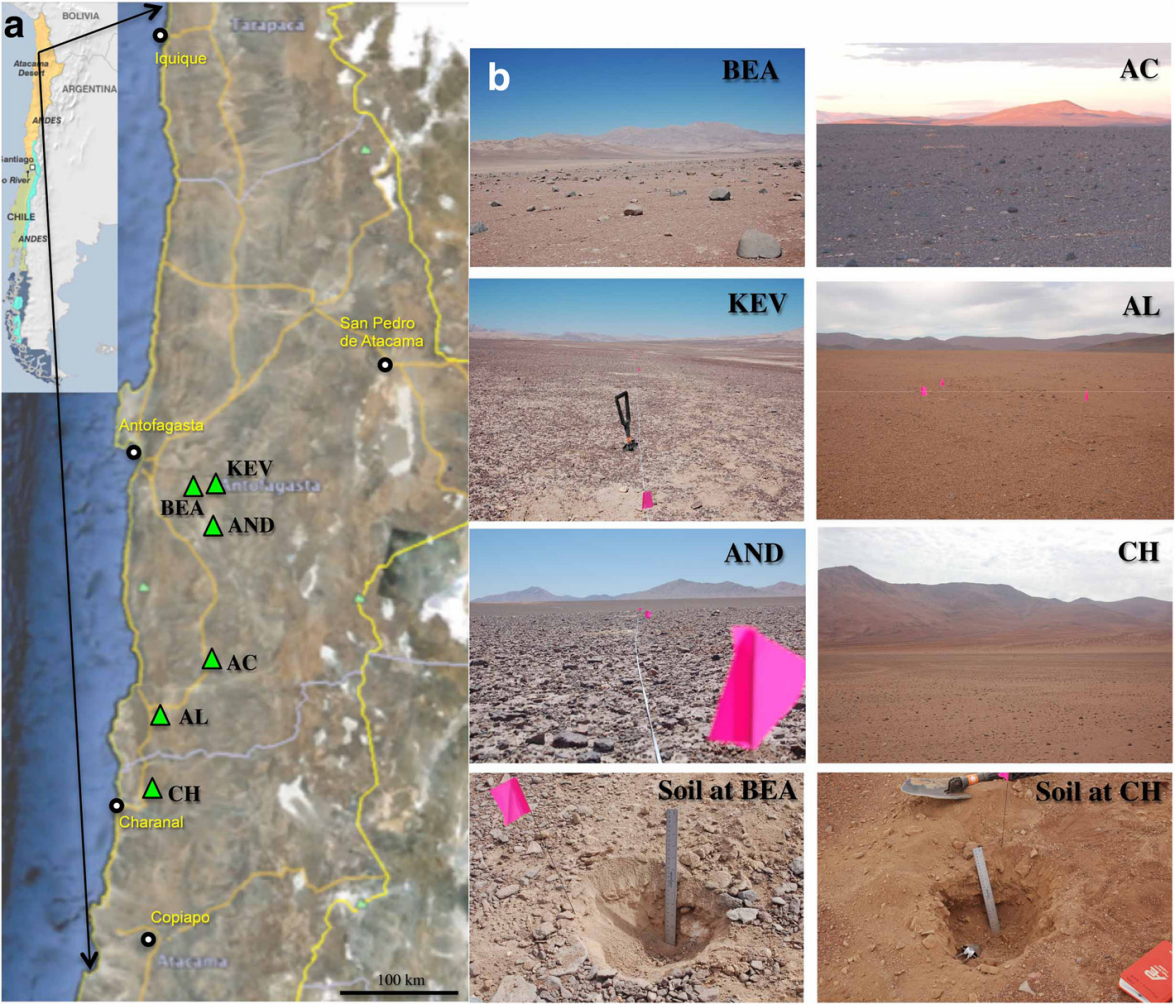
**Sampling site locations. (a)** Relief map of northern Atacama Desert, Chile, with key field locations marked by green triangles; **(b)** photos of Kevin Garden (KEV), Bea Hill (BEA), Andrew Garden (AND), Aguas Calientes (AC), Altamira (AL), and Chañaral (CH) sampling locations.

Soil samples were collected in the hyper-arid core of the Atacama Desert and along a 237-km North-South transect (Figure 1). The three locations in the hyper-arid core, Kevin Garden (KEV) (GPS: S24°01.943’, W069°42.257’; elevation 1,062 m above sea level), Bea Hill (BEA) (GPS: S24°05.110’, W069°59.619’; elevation 1,003 m above sea level), and Andrew Garden (AND) (GPS: S24°25.963’, W069°41.096'; elevation 1,506 m above sea level) were sampled in 2009 along two perpendicular transects (A and B), every 10 m (from 0 to 50 m), and at the following soil depths: 0, 5, and 10 cm. Samples at each location along the North-South transect, Bea Hill (BEA), Aguas Calientes (AC) (GPS: S25°15.466’, W069°50.924’; elevation 1,888 m above sea level), Altamira (AL) (GPS: S25°41.114’, W070°16.516’; elevation 936 m above sea level), and Chañaral (CH) (GPS: S26°09.839’, W070°17.105’; elevation 630 m above sea level) were collected in 2011, 1 m apart and in a triangle (A, B, and C), at the following soil depths: 0, 5, and 10 cm. Open soil samples were collected using aseptic techniques and stored in sealed sterile Whirl-Pack bags at room temperature during shipping (5 days) and then -20°C for all molecular analyses. A subset of the samples was stored at -4°C for mineralization experiments.

Soil total elemental analysis was performed by ICAP spectrometry following HNO_3_/HClO_4_ digestion (method 2021; Cornell Nutrient Analysis Lab, Ithaca, NY, USA). Total carbon and total organic carbon (TOC) were determined by total dry combustion with a LECO CNS-2000 Carbon Analyzer (Cornell Nutrient Analysis Lab, Ithaca, NY, USA). Soil measurements, pH, conductivity, water content, and texture were performed according to protocols described in the Standard Procedures for Soil Research in the McMurdo Dry Valleys LTER available through the MCM LTER public database at http://www.mcmlter.org.

Climate data, air temperature, and air relative humidity (RH) were collected *in situ* during the 2009 and 2011 sampling expeditions. Historical climate data were obtained from the literature [2,23,26].

Cell counts were carried out with DAPI as previously described [27].

### DNA extraction, PCR amplification, and sequencing

Total genomic DNA was extracted from soil samples using the PowerSoil DNA Isolation kit (MoBio Laboratories Inc., Solana Beach, CA, USA) following the manufacturer’s instructions. All sample manipulations and nucleic acid extractions were carried out in a laminar flow hood (AirClean Systems, Raleigh, NC, USA) and all materials and reagents were either filter sterilized, autoclaved, or UV-irradiated to prevent contamination. For 454 pyrosequencing, genomic DNA was amplified using the barcoded Universal primers 27 F (bacterial)/4Fa (archaeal) and 534R for the V1-V3 hypervariable region of the 16S rRNA gene. The amplification reaction mixture (25 μL) contained 200 μM deoxynucleoside triphosphates (dNTPs) each, 0.3 μM (each) primer, 1-5 ng/μL of DNA template, 0.02 U/μL of Phusion High-Fidelity DNA polymerase (New England BioLabs, Ipswich, MA), 1 × Phusion PCR buffer HF, 0.5 mM MgCl_2_, and 3% DMSO. PCR conditions were one initial step of 30 s at 98°C, followed by 25 cycles of 10 s at 98°C, 15 s at 55°C, and 15 s at 72°C, and with a final step of 10 min at 72°C, using a T3000 Thermal Cycler (Biometra, Horsham, PA, USA). Amplicons were purified with the AMPure Kit (Agencourt, Beckman Coulter Genomics, Danvers, MA, USA), and equimolar amounts (100 ng) of all amplicons were mixed in a single tube and sequenced by 454 pyrosequencing using a Roche GS-FLX sequencing system (Roche-454 Life Sciences, Branford, CT, USA) by the Genomics Resource Center (GRC) at the Institute for Genome Sciences (IGS), University of Maryland School of Medicine using protocols recommended by the manufacturer as amended by the GRC.

### Processing of pyrosequencing data and analysis

The 454 sequences were processed using the QIIME package (v1.6.0) [28]. Sequences were de-multiplexed by binning sequences with the same barcode and primer sequences in QIIME. Similar sequences with <3% dissimilarity were clustered together using USEARCH [29] and *de novo* chimera detection was conducted in UCHIME v5.1 [30]. The resulting average sequence length was 494 bp. Taxonomic ranks were assigned to each sequence using Ribosomal Database Project (RDP) Naïve Bayes Classifier v.2.2 [31], using 0.8 confidence values as the cutoff to a pre-built greengenes database of 16S rRNA sequences (Oct, 2012 vers.) [32]. Representative sequences of each OTU_0.03_ were aligned with PyNAST [33] against the Greengenes core set [34], gaps and parsimonious uninformative characters were removed, and the filtered sequences subsequently used to generate a phylogenetic tree with FastTree [35] for beta-diversity metrics using UniFrac [36,37]. Richness and diversity estimators were calculated based on OTUs with QIIME [28]. Principal Coordinate Analysis (PCoA) plots were generated with QIIME using unweighted and weighted UniFrac metrics, and Bray-Curtis distances [28,36]. Detrending of PCoA plots was also performed with QIIME [28]. The R statistical package was used to perform all statistical tests of diversity and geochemistry data [38]. The non-parametric Kruskal-Wallis one-way analysis of variance [39] was used to test differences in both diversity metrics and geochemistry between geographic locations and groupings. Least Squares Linear Regression was used to test correlation hypotheses.

### Mineralization

Soil microbial activity was assessed by monitoring the mineralization of ^14^C-acetate added to microcosms containing 5 g of soil and constructed according to [40]. Each microcosm, in triplicate with sterile controls (baked for 5 h at 160°C), was amended with a mixture of 1 mL of 10 mM sodium acetate (autoclaved and filtered sterilized) and 0.045 μCi (1,2-^14^C) acetic acid (100,000 disintegrations per min) (Perkin Elmer, SA 54.3 mCi mmol^-1^) at the beginning of the experiment and at day 47. Microcosms were incubated at 20°C without shaking or illumination. CO_2_ traps containing 0.5 mL of 1 M KOH were sampled every 1 to 7 days and radioactive counts were determined by liquid scintillation spectrometry using a Packard Tri-Carb 2200CA Scintillation Counter (Waltham, MA, USA). Mineralization was expressed as the cumulative radiolabel recovered as ^14^CO_2_[40]. For days 47 to 89, mineralization was calculated using the amount of radiolabeled substrate added at day 47.

## Results

We combined environmental factor measurements with molecular data from non-culture-based high throughput molecular methods to identify the factors underlying the structure and composition of microbial assemblages in soil samples from the driest areas of the Atacama Desert.

### Sampling locations and soil geochemical features

We focused our work on six locations along a North-South transect from the hyper-arid zone of the Atacama Desert to the north to a less arid area of the desert to the south; multiple sites were sampled within each of the six locations (Figure 1). The three Yungay locations within the hyper arid zone (KEV, AND, and BEA) have identical mean annual temperatures, relative air humidity (RH), and annual rainfall while two of the southern locations (AL and CH) have similar climates (Table 1). The Yungay region in the hyper arid zone is extremely dry and receives an average of <1 mm of rainfall each year. These locations have a mean air temperature of 16.5°C, which is similar to the other three locations, and a significantly low air RH of 36%. The CH and AL sites receive a greater amount of yearly rainfall, up to 12 mm, and the air RH is twice as high. The AC location is both a geographic and climatic intermediate between Yungay and the two southern locations.

**Table 1:** Geographical and climate data for soil sampling locations in the Atacama Desert

**Location**	**Latitude (S)**	**Longitude (W)**	**Elevation (m above sea level)**	**Mean annual temp (°C)**	**Mean annual relative air humidity**	**Mean annual rainfall (mm)**
KEV	24° 01.943’	069° 42.257’	1,062	16.5	36	<1
BEA	24° 05.110’	069° 59.619’	1,003	16.5	36	<1
AND	24° 25.963’	069° 41.096'	1,506	16.5	36	<1
AC	25° 15.466'	069° 50.924'	1,888	17.6	48	4.7
AL	25° 41.114’	070° 16.516’	936	16.4	70	12
CH	26° 09.839’	070° 17.105’	630	16.4	70	12

Locations KEV, BEA, and AND are found in the Yungay hyper-arid zone.

**Figure 2:**
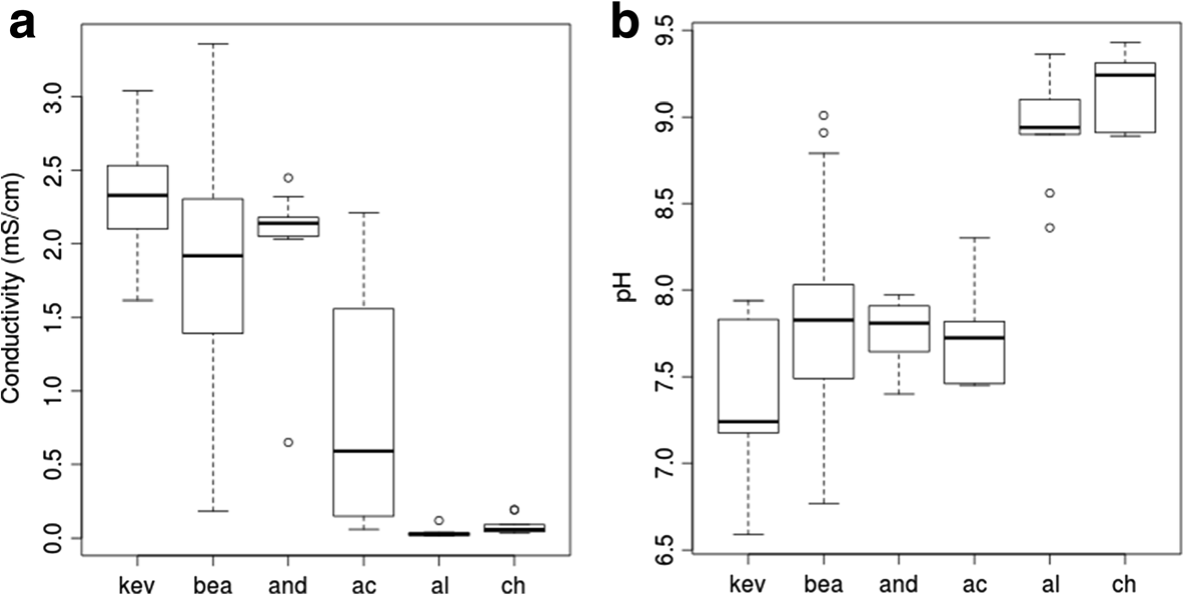
**Geochemical features of sampling locations (*n*** **= 68). (a)** Boxplots for conductivity and **(b)** boxplots for pH.

Analysis of the soil geochemistry of 68 samples (KEV, 16 samples; BEA, 18 samples; AND, 11 samples; AC, 5 samples; AL, 9 samples; and CH, 9 samples) revealed trends among and within sampling locations (Table 2; Additional file 1: Table S1). Using the non-parametric Kruskal-Wallis test for one-way analysis of variance we identified significant differences between locations in terms of pH (H(5) = 42, *P* <0.001), and conductivity (H(5) = 46, *P* <0.001); these differences were not related to soil depth across all samples (depths from 0 to 10 cm were sampled at all locations). Of the 18 BEA samples, 15 were from 2008 and three were from 2011. There was no statistical difference in conductivity between these groups (t (t-test statistic) = -1.39; *P* value = 0.27), but the 2011 samples had a higher mean pH than the 2008 samples (t = -9.35; *P* value <0.001). Partitioning the distributions of conductivity values for each geographic location separated the three Yungay locations (KEV, BEA, and AND) with higher conductivity values from the three southern locations (AC, AL, and CH) with lower conductivity values (Figure 2a). With pH values, there is a distinct separation between the two southernmost locations (AL and CH) with higher pH values from the other four locations (KEV, BEA, AND, and AC) with lower average pH values (Figure 2b). We found a significant and direct inverse relationship (β = -0.52, *P* <0.001) between pH and conductivity values (Table 2).

**Table 2:** Mean soil geochemical properties, composition, and cell counts for each sampling location in the Atacama Desert

**Location**	**pH**	**Conductivity mS.cm**^**-1**^	**Ca mg.kg**^**-1**^	**S mg.kg**^**-1**^	**Na mg.kg**^**-1**^	**Sand %**	**Silt %**	**Clay %**	**Texture**	**TC %**	**TOC %**	**Cell count 10**^**3 **^**cells.g**^**-1 **^**soil**
KEV	7.4	2.3	34186.3	24470.4	1944.5	70	13.8	16.3	Sandy loam	0.08	<0.01	3.1 (±2.5)
BEA	7.6	1.7	21308.2	9015.2	3233.1	85	6	9	Loamy sand	0.04	<0.01	6.7 (±4.3)
AND	7.8	2	33525.9	25218.2	2248.9	76	8	16	Sandy loam	0.16	<0.01	2.7 (±2.3)
AC	7.7	0.86	27244.9	12822.1	2908.6	87	5	8	Loamy sand	nd	nd	511 (±10.1)
AL	8.9	<0.1	9631.5	3.2	3498.3	93	5	2	Sand	nd	nd	24.5 (±3.1)
CH	9.2	0.1	6013.7	20.3	2928.5	83	15	2	Loamy sand	nd	nd	101 (±12.8)

nd: not determined; TC: total carbon; TOC: total organic carbon.

Soil pH, conductivity, and calcium/sulfur elemental composition were also significantly correlated across the dataset (Additional file 1: Tables S1 and S2). We found a positive linear correlation (β = 0.94, *P* <0.001) across locations between the sulfur and calcium elemental compositions - the components of gypsum and anhydrite (CaSO_4_, +/-2H_2_O). The calcium and sulfur values we measured for each location correlated strongly with average soil conductivity for each location. The soil in the AL and CH locations had lower amounts of elemental calcium and sulfur than the soil of the other four locations. The AC location shared geochemical features with both the southern and northern locations; while it has a low average pH (7.75, s (standard deviation) = 0.3) and high levels of calcium and sulfur like the three Yungay locations, a significantly lower average conductivity of 0.86 mS (millisiemens)/cm (s = 0.87) was recorded. The variation in conductivity at AC was higher than any other location (Figure 2). The lowest variations in soil conductivity were observed in the AL and CH samples (s = 0.03, s = 0.06). Soil composition for most samples was sandy loam and we measured extremely low levels of total organic carbon (Table 2; data for 2009).

Within each location, we found some variability for conductivity and pH values. There was a significant positive increase in pH (t = 4.55; *P* = 0.0002) with depth when the data were parsed for each sampling site, but no similar change in conductivity (Additional file 1: Table S10). Non-stochastic patterns of geochemistry across transects of sampling sites were not observed.

### Molecular characterization of the soil microbial communities

We characterized the soil microbial communities from several geographic locations of the Atacama Desert using pyrosequencing of barcoded 16S rRNA gene PCR amplification products using primer sets for Bacteria and for Archaea. No Archaea were found in any of the samples despite multiple attempts at PCR amplification using specific archaeal primers (4Fa and 338R [41]) with and without nested primers. We removed biases from sequencing efforts, by generating taxonomic data rarefied to both 200 and 1,000 sequence counts using QIIME [28]. This resulted in the analysis of 139,549 sequence reads from 68 soil samples (as described above) with at least 200 sequences and 48 of these samples had at least 1,000 sequences. This dataset represents an unprecedented level of detail for these communities. Assignment of Operational Taxonomic Units (OTUs_0.03_), defined as sharing at least 97% sequence similarity, resulted in a number of OTUs_0.03_ ranging from 69 to 332 (1,000-rarefaction level) per sample, revealing that communities with wide ranges of richness inhabit the soil of the Atacama Desert (Table 3). Diversity metrics at the 200-sequence level were lower than the same metrics at the 1,000-sequence level but comparative trends between samples were similar at both levels (Additional file 1: Table S3). Absolute diversity metrics were reported at the 1,000-sequence level for maximum coverage, while correlation analyses were performed at the 200-sequence level in order to include as many samples as possible. The slope of the rarefaction curves indicated that a large fraction of the community was sampled at the 1,000-sequence level (Additional file 1: Figure S1). While new OTUs were still being sampled at the 200-sequence level, the trend across the samples was conserved.

**Table 3:** **Observed richness and diversity indices for soil samples based on 16S rRNA gene sequence assignments with a 97% sequence similarity threshold, rarefied to 1,000 sequence reads (*n*** **= 48)**

**Samples**	**OTU**_**0.03 **_**observed richness**	**Shannon index estimated diversity**	**Faith’s phylogenetic diversity index**	**Chao 1 diversity estimate**	**Pielou’s evenness index**
KEV	69	2.6	3.1	134	0.42
BEA	99	4.3	5.7	157	0.64
AND	83	3.8	5.3	164	0.59
AC	163	5.9	9.6	244	0.80
AL	330	7.5	15.9	508	0.89
CH	282	7.0	14.5	396	0.85

Analysis using datasets of equal size subsampled at 1,000 sequence reads; average of 10 iterations.

OTU, operational taxonomic units.

**Figure 3:**
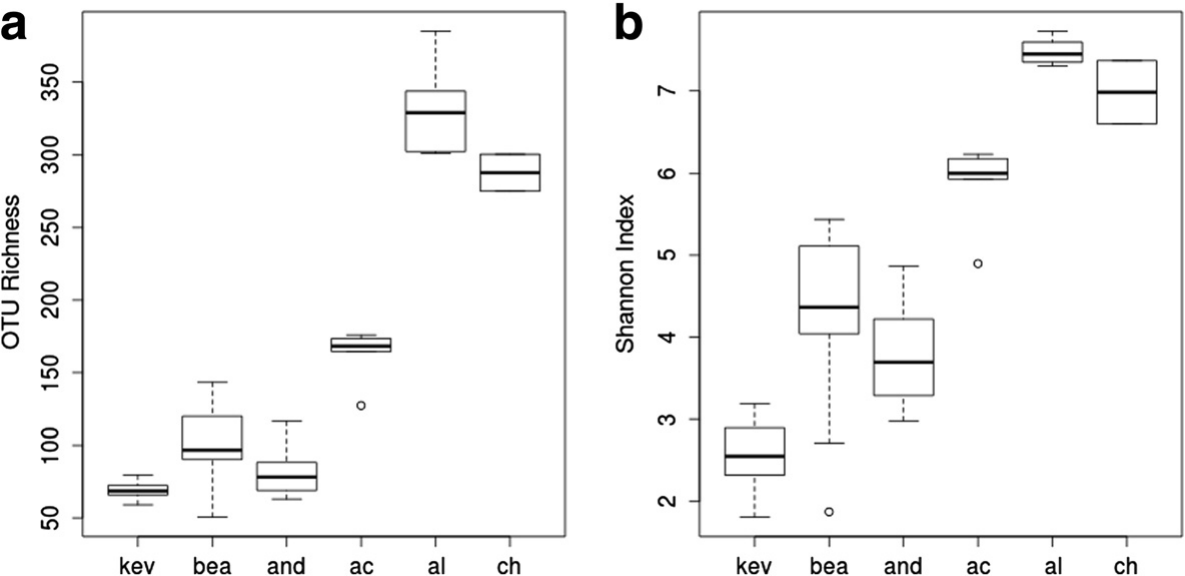
**Alpha diversity metrics for each sampling location, rarefied to the 1,000-sequence level (*n*** **= 48). (a)** Boxplots of OTUs_0.03_ richness and **(b)** boxplots of the Shannon diversity index, both at the 97% sequence similarity threshold.

**Figure 4:**
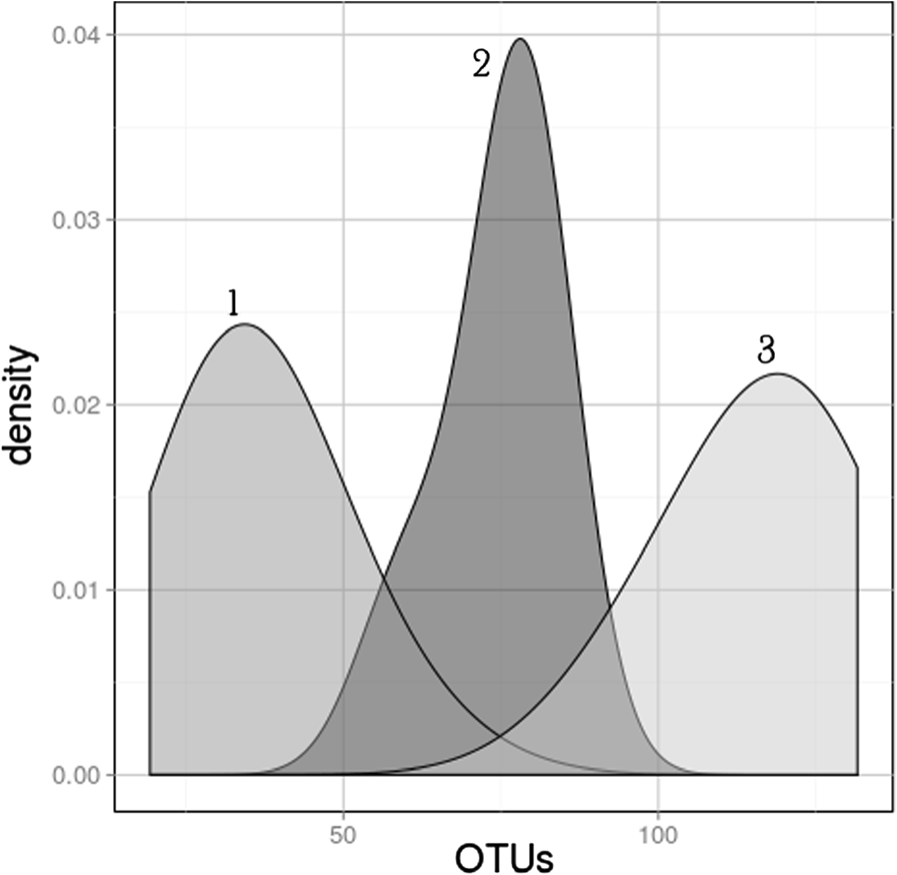
**A density plot of observed OTU**_**0.03 **_**richness, rarefied to the 1,000-sequence level (*n*** **= 48), comparing the (1) northern (KEV, BEA, AND) locations, the (2) central (AC) location, and the (3) southern (AL, CH) locations.** The vertical height of each grouping is the continuous probability that each group contains a sample with the given richness.

Several descriptive and predictive alpha metrics of diversity were calculated for each sample using QIIME [28] (Table 3). Ten iterations of the analyses were performed (1,000 sequence reads each) and the alpha diversity data from each were averaged. Across all samples, the average percent deviation from the mean was 4.1% for OTUs_0.03_, 1.6% for Shannon, and 14.0% for ChaoI. There was no statistical difference in richness between the BEA samples collected in 2008 (15 samples) and in 2011 (3 samples) (t = 1.63; *P* = 0.185). A large number of observed OTUs_0.03_ were either singletons or doublets with abundance of those ‘rare’ OTUs_0.03_ (singletons + doublets) similar across all samples (Additional file 1: Table S4). We found there were three distinct clusters of locations based on alpha diversity; the northern Yungay locations (BEA, KEV, AND) with the lowest diversity, the central AC location, and the southern locations (CH, AL) with the highest levels of diversity (Table 3, Figure 3). Kernel probability density estimations of richness, which describe the relative likelihood of a sample within a distribution having a given richness, were generated for each of the three aforementioned clusters. Comparing the probability densities between the clusters established three visually distinct geographic distributions (Figure 4). Variation in alpha diversity between locations was significant and greater than variation within each location. The KEV location had the lowest mean diversity, and a non-parametric Kruskal-Wallis ANOVA test showed that it was significantly lower in observed OTUs_0.03_ than the BEA and AND locations (*P* <0.001 and *P* <0.001, respectively), both of which in turn were significantly lower than the AC site (*P* <0.001; *P* <0.001). The AC location also contained significantly fewer observed OTUs_0.03_ than the CH and AL locations (*P* <0.001; *P* <0.001, respectively). The range of alpha diversity metrics within each site was small with few outliers (Figure 3).

To test correlation between abiotic factors and soil microbial diversity metrics, we performed tests of Least Squares linear regression on the alpha diversity metrics generated at the 200-sequence level (*n* = 68). We found that conductivity and pH both significantly correlated with community richness, predicted diversity, evenness, phylogenetic diversity, and Shannon diversity across all locations (Additional file 1: Table S5). pH correlated directly with richness (*P* <0.0001) and conductivity correlated inversely with richness (*P* <0.001). Although the AC location had a slightly lower mean pH than either BEA or KEV, it had a significantly higher distribution of alpha diversity metrics (Table 3). The same correlations proved significant at the 1,000-sequence level (Additional file 1: Table S6). There were no significant relationships between both OTU_0.03_ count and Chao1 predicted diversity with soil depth at either the 1,000 or the 200 level. Location climatic data were significantly correlated with all metrics of diversity (Additional file 1: Table S7). The climatic data are location-specific as opposed to sample-specific, and the correlation demonstrates the difference in diversity levels between the hyper-arid northern sites and the southern sites. Mean air relative humidity (RH), mean air temperature, and rainfall correlated directly with observed OTUs_0.03_ (*P* <0.001). Within locations, diversity did not correlate with sample depth or with sampling site position and this included the sampling-transects for each of the three northern locations, KEV, BEA, and AND.

**Figure 5:**
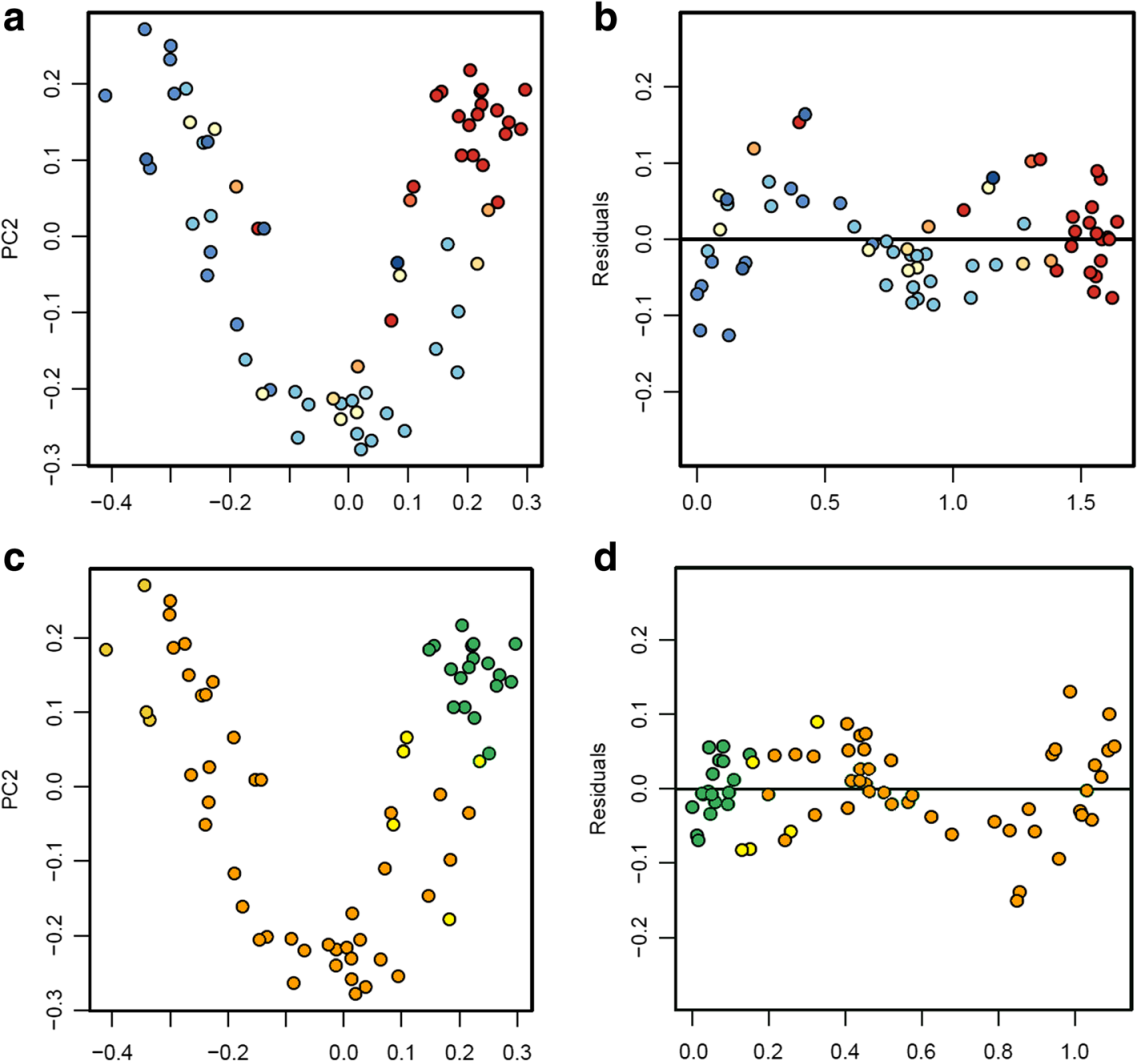
**Unweighted Unifrac principal coordinate analyses (PCoA) of samples at the 200-rarefied level (*n*** **= 68), calculated with QIIME. (a)** PCoA color-coded by conductivity values (low conductivity: red, average: white, high: blue), and **(b)** the same plot detrended with QIIME. **(c)** PCoA color coded by relative air humidity values (high humidity: green, average: yellow, low: orange), and **(d)** the same plot detrended with QIIME.

Beta diversity metrics were calculated to assess community similarity and to evaluate the effect of environmental and geographic metadata on the observed variations in diversity between sampling sites. Clustering analysis of rarefied 200-sequence (*n* = 68) for Bray-Curtis distances showed that communities from each location were more similar to each other than to communities from other locations and that communities from north/central and south locations clustered separately (Additional file 1: Figure S2). Average pairwise comparisons between all samples using unweighted UniFrac distances indicated that a community at any sampling site was more similar to another community from the same geographic location than to any other location (Additional file 1: Table S8). These results, together with non-parametric ANOVA test of alpha diversity metrics, indicated that each geographic location has distinct microbial assemblages. Principal coordinate analyses (PCoA) were performed on the rarefied 200-sequence (*n* = 68) unweighted UniFrac distance matrix using QIIME [28] (Figure 5a and 5c). Axis 1 of the PCoA plot explained 16% of the variation, and axis 2 explained 11% of the variation. When color-coded by conductivity values and air RH values, a visual separation of clusters is evident. Conductivity correlated strongly with both axis 1 of the unweighted UniFrac PCoA (Spearman’s R: 0.79; *P* <0.0001) (Figure 5a) and axis 1 of a similar weighted UniFrac PCoA (Spearman’s R: 0.64; *P* <0.0001) (Additional file 1: Figure S3a). Mean air RH also correlated strongly with axis 1 of the unweighted UniFrac PCoA (Spearman’s R: -0.66; *P* <0.0001) (Figure 5c) and axis 1 of the weighted UniFrac PCoA (Spearman’s R: -0.63; *P* <0.0001) (Additional file 1: Figure S3c). As for pH, we found a good correlation with axis 1 of the unweighted UniFrac PCoA (spearman’s R: -0.78; *P* <0.0001), but a lower correlation with axis 1 of the weighted UniFrac PCoA (spearman’s R: -0.44; *P* <0.001). In order to better represent the differences between samples in a linear fashion, the plot was detrended using QIIME and color-coded (Figures 5b and 5d). Sample depth did not correlate with either axis of an unweighted UniFrac PCoA. Similar trends were observed using data rarefied at the 1,000-level sequence reads (Additional file 1: Figure S4).

### Phylogenetic diversity of soil microbial communities

**Figure 6:**
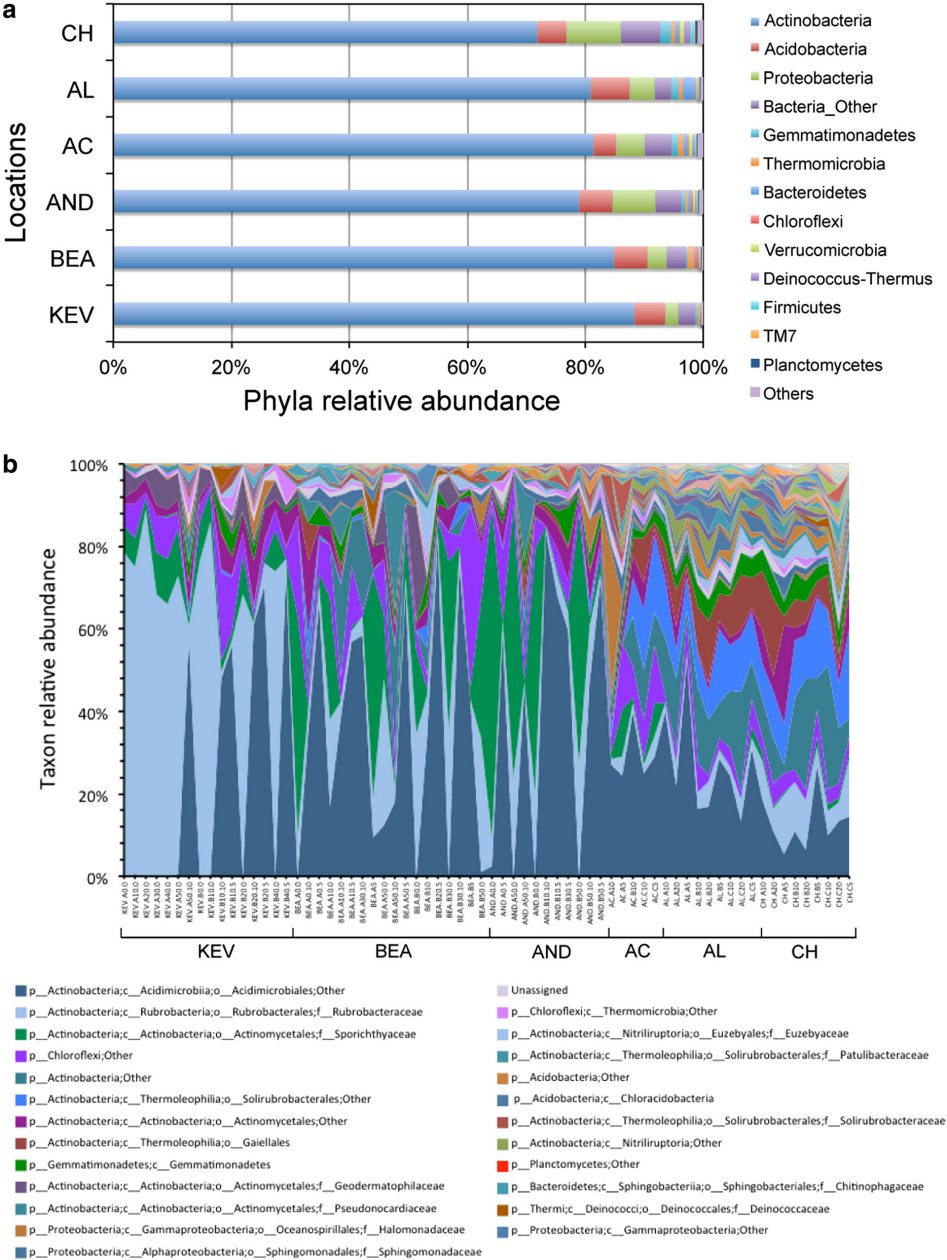
**Relative abundance of major taxonomic groups in all Atacama soil samples based on environmental 16S rRNA gene sequences. (a)** Phyla relative abundance; phyla with <1% abundance are represented in ‘others’; **(b)** relative abundance of order/families (>10 counts summed across all samples); first 25 taxa are described in the figure legend; a list of all taxa is in (Additional file 1: Table S9). Analyses were performed using samples at the 200-rarefied level (*n* = 68).

Sequence reads were classified with the Ribosomal Database Project Classifier at 0.8 confidence threshold [31] in order to assess the phylogenetic distribution and taxonomic diversity of the soil samples. The overall phylum-level composition was similar among the six desert locations and dominated by *Actinobacteria* (72% to 88%; Figure 6a), followed by *Acidobacteria* (3.8% to 6.6%), and *Proteobacteria* (2.2% to 9.2%). Differences in phyla abundance between sample’s groups were tested using Metastats [42]. We found that the increased abundance in members of the *Gemmatimonadetes* and *Planctomycetes* in the south samples (AC, AL, and CH) when compared to the north samples (KEV, BEA, and AND) was statistically significant (*P* >0.001; q = 0.02). No archaeal sequences were found and 2.8% to 6.7% of the bacterial sequences were unassigned.

Relative abundance of taxa at the order/family level (Figure 6b) greatly illustrated the change in community structure and composition revealed by analyses of alpha and beta diversity. Members of the *Actinobacteria* mostly belong to a small number of orders including *Rubrobacterales*, *Actinomycetales*, and *Acidimicrobiales* (Figure 6b). While *Rubrobacterales* were dominant in the KEV location, a shift toward *Acidimicrobiales* and *Actinomycetales* was observed for the BEA and AND locations. At the taxonomic level, the most southern locations (AC, AL, and CH) were more diverse with members of the *Acidimicrobiales, Rubrobacterales* but also of the *Gemmatimonadetes, Bacteroidetes,* and a number of families from the *Thermoleophilia* (class of *Actinobacteria*) [43]. The evenness of these later communities, represented by the Pielou’s evenness index, was notably higher than that of the northern communities (Table 3) and expressed by a larger number of members per taxonomic group (Figure 6b). While a number of *Chloroflexi* taxa were found across all soil samples, very few members of the *Firmicutes* were detected.

### Soil metabolic activity

**Figure 7:**
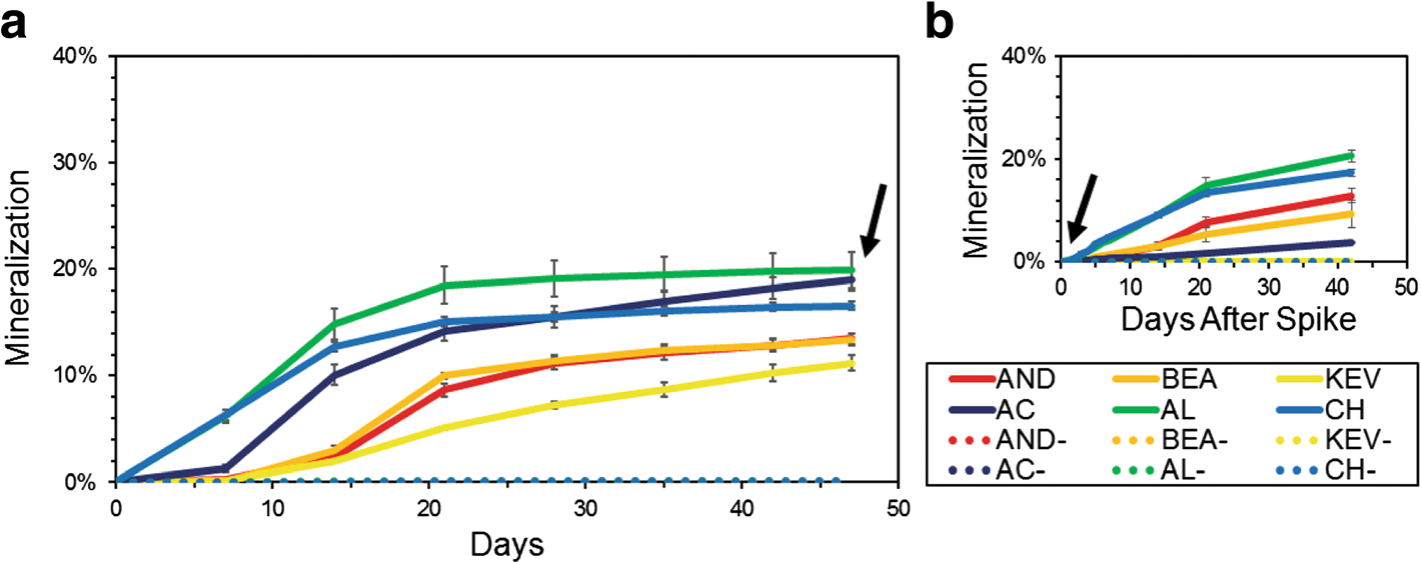
**Mineralization of (1,2-**^**14**^**C) acetic acid at 20°C in microcosms with Atacama soil samples.** Each point represents the mean cumulative mineralization expressed as the cumulative % of radiolabel recovered as ^14^CO_2_ from triplicate assays including sterile controls (dashed lines). **(a)** Radiolabeled acetic acid (0.045 μCi) was added at the beginning of the experiment and **(b)** again at day 47 to the same microscosms. Black arrows: second addition of radiolabel substrate. Error bars are standard error of the mean.

Mineralization of radiolabeled acetate was detected in all soil samples but rates were notably different (Figure 7). Samples from the south locations showed mineralization within 7 days and reach maxima of 18% to 21% of acetate mineralized to CO_2_ in 21 days, whereas we observed a lag time for mineralization with samples from the north locations and lower mineralization maxima. A second addition of substrate at 47 days resulted in a rapid restart of mineralization - within 1 day - for all samples with the exception of AC (Figure 7). Controls did not show any activity for the 90-day experiment.

## Discussion

The Atacama Desert presents a unique physiography, a range of hyper-arid conditions, climate regimes, and geology within a relatively small region, providing a number of unique habitats for microbial communities [24,25]. Of particular relevance is a steep rainfall gradient along a North-South transect between 23ºS and 29ºS, where precipitation increases from <1 mm year^-1^ to >40 mm year^-1^[2,23]. The Atacama rainfall gradient represents a unique natural setting to study how life responds to increasing water stress towards the hypothetical dry limit of life. We conducted a high-resolution sampling of near surface soils (0-10 cm) and applied a combination of geochemistry and molecular data to characterize the microbial community along the rainfall gradient, and to determine the environmental factors shaping the community structure in these soils.

Geochemical analyses of our study sites revealed a high degree of heterogeneity with the greatest variability in soil mineralogy between geographical locations. The southernmost soils were more alkaline than the other soils, which might reflect a greater influence from salts of marine origin [44,45]. In contrast, more inland salt deposits in the Atacama are thought to be the result of eolian dispersion from local salars [44].

Our molecular data provided a good coverage of the soil microbial diversity, underlining the low microbial diversity of the Atacama soil microbiome. Similarly low numbers for OTUs_0.03_ richness were reported for comparable studies of Atacama soils at the hyper-arid margin [16] and for several locations in the Antarctica Dry Valleys [46]. Alpha diversity metrics showed stochastic variations at multiple sampling sites within each geographic location, but significant differences were found between locations. Analyses of beta diversity indicated that communities from each geographic location were structurally and phylogenetically distinct.

Changes in soil microbiology along the North-South transect were correlated with water availability, described as air mean RH and mean annual rainfall. Changes were also inversely correlated with soil conductivity, a proxy for water availability (that is, more salt, less water) [47]. Microbial diversity, community structure, and rates of metabolic activity were clearly distinct between the driest localities and the wettest ones, with an apparent intermediate transition zone (AC) located between S25º0’ and S25º30’. Lowest diversity was found in the northern samples where water availability reaches minimum values. These soils also had relatively high salt contents associated with high levels of Na, Ca, and S, and the presence of gypcrust close to the soil surface (within 10 cm), indicating persistent aridity over geological time [48]. Microbial diversity was significantly higher at the southernmost sampling localities where mean annual rainfall was one order of magnitude higher and near-surface soils were likely wetted yearly. The higher level of precipitation experienced by the southern soils was substantiated by low salt contents as the result of leaching toward higher depths [48,49].

Soil heterotrophic bacteria commonly encountered in arid environments were shared between all soil samples and included members of the *Rubrobacterales*, *Actinomycetales*, and *Acidimicrobiales*[16,50]. The abundance of the various taxonomic groups varied significantly between soil locations. Radiation tolerant and desert dwelling bacteria from the *Rubrobacterales* were most abundant in the driest locations while the southern locations displayed a number of *Thermoleophilia* families, including *Solirubrobacteraceae* and *Patulibacteraceae*. These taxa, closely related to the *Rubrobacteraceae*, are found in biological soil crusts [51] and have been reported in less arid soils of the Atacama Desert [16]. *Chloroflexi* were found across all soil locations and have been observed in hypolithic and various soil communities of the Atacama Desert [16,52-54]. No archaeal sequences were found as was reported for Antarctic soils of similar composition [46,55].

Using soil microcosms amended with radiolabeled acetate we detected metabolic activity in all tested samples, indicating a viable portion of the soil community in all localities. These are conventional methods typically used to detect metabolic activity in extreme environments such as at subzero temperatures and in hyper-arid deserts [8,56-59]. Different experimental settings make it difficult to compare our results with previous experiments using Atacama soil [8,57]; however, similarly to Quinn et al. [57], we observed a resumption of CO_2_ production after a second injection of substrate. Our mineralization rates were consistent with studies using Canadian high Arctic soil samples, taking into consideration differences in incubation temperatures [40,60,61]. Detectable mineralization rates in the northernmost Atacama samples were observed after 7 days of incubation, while the southernmost samples mineralized faster and in higher amounts. These trends could be due to a difference in cell abundance, resulting in smaller rates of mineralization and smaller consumption of labeled substrate in the northernmost samples. Alternatively, they could be explained by a difference in the physiological state of soil micro-organisms, with a rapid activation in the southernmost samples that witness yearly rainfall, and a slow activation in the northernmost samples that witness decadal rainfall. While estimated cell abundances vary by up to two orders of magnitude between the northernmost and the southernmost samples (Table 2), mineralization rates and net amounts were similar between AL (2.4×10^4^ cells g^-1^ of soil) and CH (1×10^5^ cells g^-1^ of soil), suggesting that both cell abundance and physiological state might be significant in explaining the different rates of mineralization. Soil micro-organisms at the dry end of our sampling transect are challenged by severe oligotrophic conditions, as illustrated by the extremely low TOC and total nitrogen levels in these soils (Table 2 and [11,16,62]), and it is therefore likely that both extreme water stress and restricted access to organic substrates are limiting factors for their *in situ* activity and growth. Determining the state of dormancy and the rate at which metabolic activity is recovered in Atacama soil communities will require careful isolation and in depth physiological and molecular studies of microbial strains from both the north and south locations.

Together, our results suggest that: (1) soil micro-organisms in the driest Atacama soils are in a state of stasis for most of the time, but can potentially metabolize if presented with liquid water for sufficient time; and (2) there is, or has been, a degree of selection on the soil microbial communities in response to environmental conditions. Correlation with water availability and soil salt content revealed that these factors are potentially the drivers for the variations in diversity observed in the soil, and likely the drivers for selection. One possible source of water in this extremely dry environment could be the deliquescence of soil salts such as NaCl, similar to micro-organisms inhabiting halite nodules in ancient saltpans within the same hyper-arid region [63,64]. A similar mechanism was also suggested for halite- and perchlorate-rich soils, 2-m-deep in the Atacama subsurface [65]. The soils we analyzed did not contain nearly as much salt as the hypersaline subsurface described by Parro et al. [65], suggesting that near-surface soil (up to 10 cm) of the hyperarid core, because of its loose structure and limited amount of hygroscopic salts, presents little potential for water retention. Only rain events with 2 mm of rainfall or more were shown to generate free water in the top few centimeters for this type of soil (measured by the increased voltage between two electrodes 5 mm apart [66]). These events are rare and short-lived, and seemingly linked to el Niño decadal cycles [2]. Therefore, it appears that conditions for metabolic activity and selection in these hyper-arid soils only occur during infrequent rain events over decades or longer. This would explain the very long residence time of organic carbon in these soils (c.a. 10^4^ yr, [49]), and the long-term preservation of labile organic compounds such as amino acids [67].

## Conclusion

Our work suggests that soil micro-organisms in the hyper-arid core of the Atacama Desert are still viable, but possibly at the limit of survival. It further emphasizes the hypothesis that in extremely dry environments, habitability becomes heterogeneous and needs to be evaluated in terms of the physicochemical properties of different substrates potentially ‘habitable’. The meager biological content and metabolic activity in the hyper-arid soils of the Atacama is in stark contrast with other substrates found in the same hyper-arid region, such as calcite, gypsum, and ignimbrite rocks [19-21], and the interior of hygroscopic salts (halites) that can sustain diverse and metabolically active communities by way of salt deliquescence [63,64,68]. Similarly, hygroscopic salts on Mars could have become one of the last refuges for life on the planet, long after soils became uninhabitable [69]. Hence, a rigorous search for life on Mars demands the study of a diversity of substrates, and particularly those that are known to sustain life in extremely dry environments on Earth.

## Availability of supporting data

The datasets supporting the results of this article are available National Centre for Biotechnology Information Sequence Read Archive under SRA accession number SRA091062, and study accession number SRP026010, BioProject ID PRJNA208226.

## Abbreviations

AC: Aguas Calientes; AL: Altamira; AND: Andrew Garden; ANOVA: Analysis of variance; BEA: Bea Hill; CH: Chañaral; ICAP spectrometry: Inductively coupled argon plasma spectrometry; KEV: Kevin Garden; OTU: Operational taxonomic unit; PCoA: Principal coordinate analysis; RH: Relative humidity.

## Competing interests

The authors declare that they have no competing interests.

## Authors’ contributions

JDR, AFD, and CPM conceived of the project. JDR and AFD collected the samples. CKR conducted nucleic acid extractions, PCR amplification, and processed the samples for sequencing. WFF supervised sequence data generation. ACC, JDR, BJ, and CKR carried out the sequence and statistical analyses. TB performed the mineralization experiments. TB and ACC performed geochemistry analyses. ACC and JDR wrote the manuscript. All authors read and approved the final manuscript.

## Supplementary Material

Additional file 1 Supporting Information. Click here for file
