# Supplementary material for: Colonization patterns of soil microbial communities in the Atacama Desert
Source: Microbiome. 2013 Nov 20;1:28. doi: 10.1186/2049-2618-1-28 (PMC3971613; doi:10.1186/2049-2618-1-28)
Supplement: Additional file 1 — Supporting Information. [file 2049-2618-1-28-S1.doc]

**Supporting Information**

**Colonization patterns of soil microbial communities in the**

**Atacama Desert**

Alexander Crits-Christoph^1^, Courtney K. Robinson^1^, Tyler Barnum^1^, Jacques Ravel^2^, W. Florian Fricke^2^, Alfonso F. Davila^3^, Bruno Jedynak^4^, Christopher P. McKay^3^, and Jocelyne DiRuggiero^1§^

**1. Supporting tables**

**Table S1** Relationships between geochemical properties of soil samples

**Table S2** Geochemical elemental composition for sampling locations

**Table S3** Observed richness and diversity indices for soil samples

**Table S4** OTU_0.03_ distribution for soil samples

**Table S5** Relationships between geochemical properties of soil samples and diversity metrics; rarefaction to 200 sequence reads

**Table S6** Relationships between geochemical properties of soil samples and diversity metrics; rarefaction to 1000 sequence reads

**Table S7** Relationships between climatic properties of locations and richness of individual samples; rarefaction to 200 sequence reads

**Table S8** Average pairwise comparisons of unweighted UniFrac distances

**Table S9** Complete legend for Fig. 6

**Table S10** Geochemical data for soil samples at each sampling locations

**2. Supporting figures**

**Fig. S1** Rarefaction plot for soil samples for observed OTU_0.03_

**Fig. S2** UPGMA tree of Brays-Curtis distances

**Fig. S3** Weighted Unifrac PCoA of soil samples at the 200-rarefied level

**Fig. S4** Unweighted Unifrac PCoA of soil samples at the 1000-rarefied level

**Table S1** Least squares linear regression of relationships between geochemical properties of soil samples. *n=68, df=67*

**Table S2** Geochemical elemental composition for each of the sampling locations

**Table S3** Observed richness and diversity indices for soil samples based on 16S rRNA gene sequence assignments with a 97% sequence similarity threshold, rarefied to 200 sequence reads (n=68)

Abbreviation: OTU, operational taxonomic units

Analysis using datasets of equal size subsampled at 200 sequence reads; average of 10 iterations

**Table S4** OTU_0.03_ distribution for soil samples (n=48)

Analysis using dataset subsampled at 1000 reads; average of duplicates

Rare OTUs_0.03_: doublets contain 2 sequence reads; singletons contain one sequence read

**Table S5** Least squares linear regression of relationships between geochemical properties of soil samples and diversity metrics. *n=68, df=66*, rarefaction to 200 sequence reads

**Table S6** Least squares linear regression of relationships between geochemical properties of soil samples and diversity metrics. *n=48, df=46*, rarefaction to 1000 sequence reads

**Table S7** Least squares linear regression of relationships between climatic properties of locations and richness of individual samples within those locations. *n=68, df=66*, rarefaction to 200 sequence reads

**Table S8** Average pairwise comparisons of unweighted UniFrac distances between samples from each location at the rarefied 200-sequence level (n=68). The distances of each sample to itself were not included in the calculation

**Table S9** Legend for Fig. 6; abundance goes from low (top) to high (bottom)

**Table S10** Geochemical data for soil samples at each sampling locations

In bold, samples from 2011; naming strategy different than from 2008

**Fig. S1** Rarefaction plot for soil samples for observed OTU_0.03_ rarefied to the 1000 sequence level (n=48).

**Fig. S2** β-diversity analysis of soil microbial communities. UPGMA tree of Brays-Curtis distances for rarefied 200-sequence reads (n=68) based on OTUs_0.03_ relative abundance with geographic location of each samples, North (KEN, BEA, AND), Central (AC), and South (AL, CH). Scale bar represents 0.05% sequence divergence. *sample from a south location in a central location cluster.


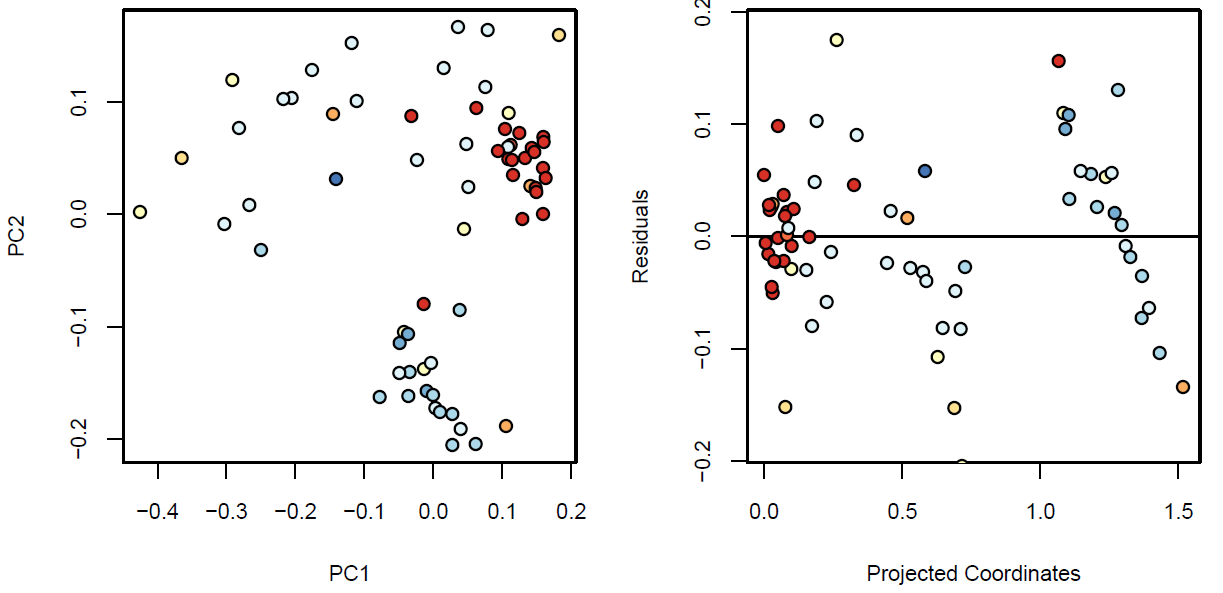

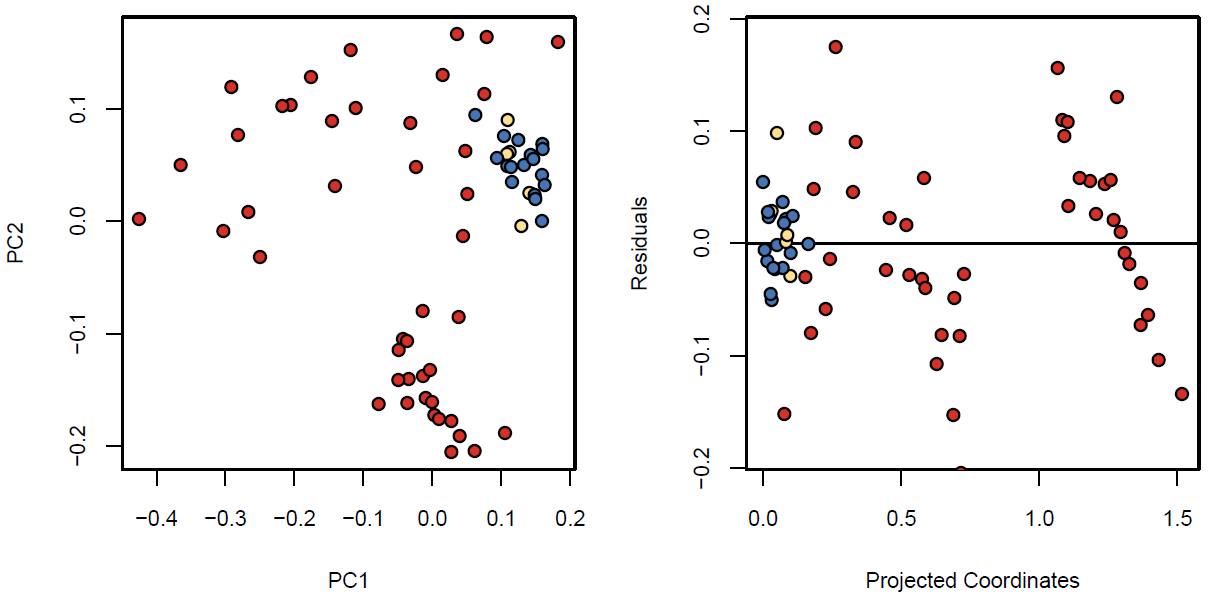


a

b

c

d

**Fig. S3** Weighted Unifrac Principal Coordinate Analyses (PCoA) of samples at the 200-rarefied level (n=48) using QIIME. (a) PCoA color coded by conductivity values (low conductivity: red, average: white, high: blue), and (b) the same plot detrended with QIIME. (c) PCoA color coded by relative air humidity values (high humidity: blue, average: white, low: red), and (c) the same plot detrended with QIIME.


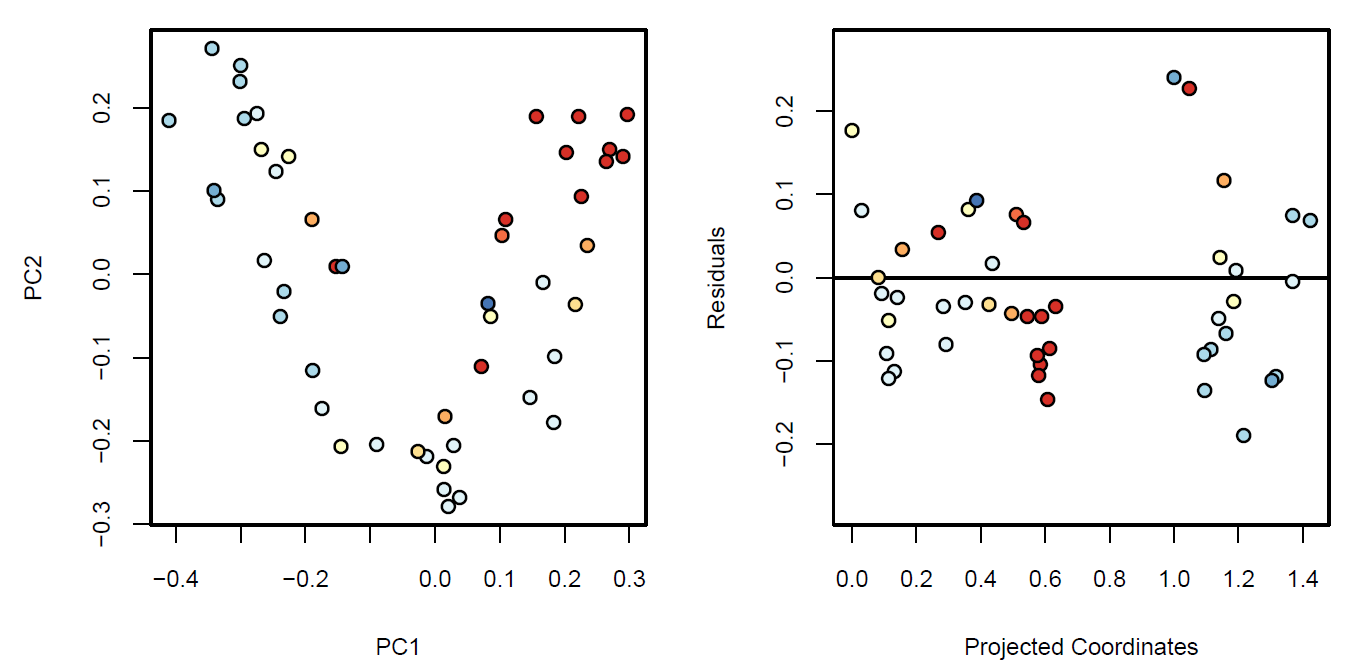

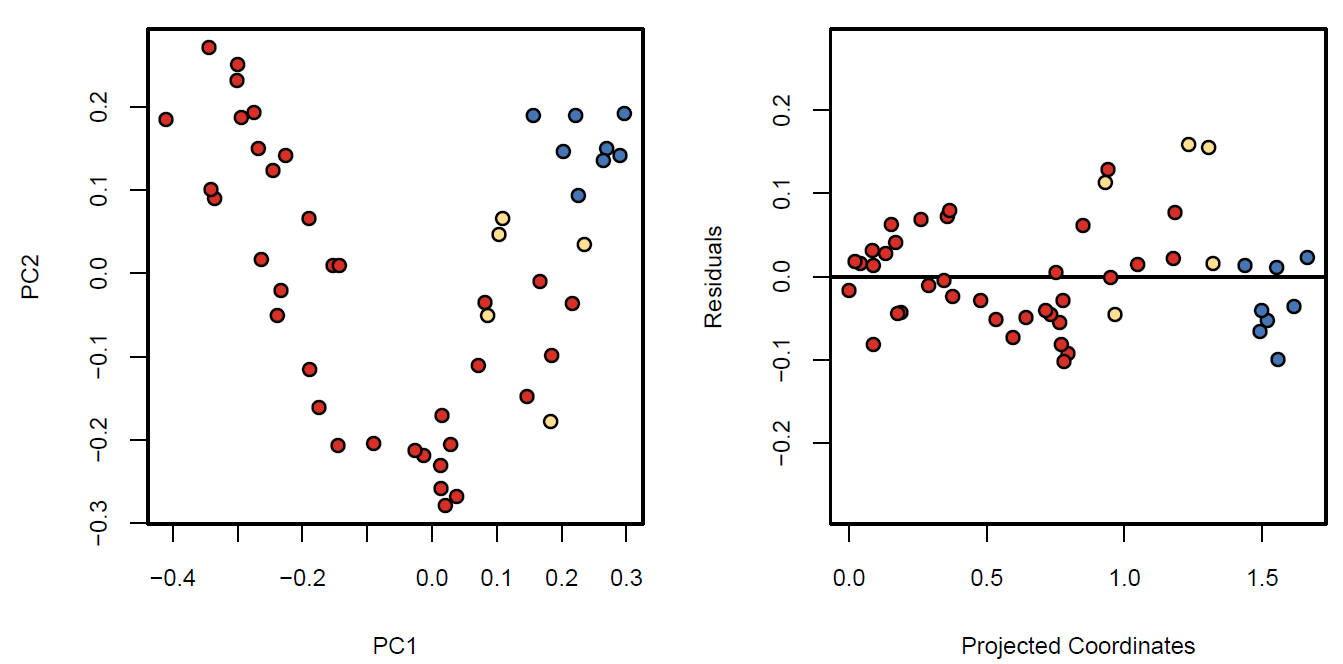


a

b

c

d

**Fig. S4** Unweighted Unifrac Principal Coordinate Analyses (PCoA) of samples at the 1000-rarefied level (n=48), calculating using QIIME. (a) PCoA color coded by conductivity values (low conductivity: red, average: white, high: blue), and (b) the same plot detrended with QIIME. (c) PCoA color coded by relative air humidity values (high humidity: blue, average: white, low: red), and (c) the same plot detrended with QIIME.
